# Osseointegration of a novel dental implant in canine

**DOI:** 10.1038/s41598-021-83700-4

**Published:** 2021-02-22

**Authors:** Lingxiao Wang, Zhenhua Gao, Yucheng Su, Qian Liu, Yi Ge, Zhaochen Shan

**Affiliations:** 1 Outpatient Department of Oral and Maxillofacial Surgery, School of Stomatology, Capital Medical University, Tian Tan Xi Li No. 4, Beijing, 100050 People’s Republic of China; 2 Department of Stomatology, Chinese Academy of Medical Science & Peking Union Medical College Hospital, No. 41 Damucang Hutong, Xicheng District, Beijing, 100032 People’s Republic of China; 3 Beijing Citident Stomatology Hospital, Beijing, 100032 People’s Republic of China

**Keywords:** Oral diseases, Preclinical research, Translational research

## Abstract

This study aimed to compare and verify the osseointegration performance of a novel implant (NI) in vivo, which could provide a useful scientific basis for the further development of NIs. Thirty-two NIs treated with hydrofluoric acid and anodization and sixteen control implants (CIs) were placed in the mandibles of 8 beagles. Micro-CT showed that the trabecular number (Tb.N) significantly increased and trabecular separation (Tb.Sp) significantly decreased in the NIs at 2 weeks. Significant differences were found in the trabecular thickness, Tb.N, Tb.Sp, bone surface/bone volume ratio, and bone volume/total volume ratio between the two groups from the 2nd–4th weeks. However, there were no significant differences between the two groups in the bone volume density at 2, 4, 8, or 12 weeks or bone-implant contact at 2 or 4 weeks, but the BIC in the CIs was higher than that in the NIs at the 8th and 12th weeks. Meanwhile, the histological staining showed a similar osseointegration process between the two groups over time. Overall, the NIs could be used as new potential implants after further improvement.

## Introduction

The design of dental implants and their various unique dental implant systems is the most active and dynamic area of research in the field of oral implantation. In 1952, the osseointegration of pure titanium was discovered and advanced by Professor Branemark. Based on a theoretical design, he developed the landmark Branemark dental implant system, which has been successfully used in the field of oral restoration^1^. Currently, researchers in various countries, especially many developed countries, have developed and launched excellent implant systems, which have been used to perform extensive fundamental research. At present, there are more than 500 kinds of dental implant systems in the world, and more than 50 kinds of dental implants are widely recognized by dentists specializing in implant dentistry and by patients with missing teeth due to the efficient and stable osseointegration of these implants^2–7^.

Evaluation of the quality of an implant is mainly concentrated on three aspects: materials, surface properties and design. The mainstream material is still pure titanium, but novel titanium alloys, such as Ti–Zr, Ti–20Nb–10Zr–5Ta, and Zr61Ti2Cu25Al12, and innovative production methods for nonmetallic materials and ceramic composites are promising candidates for future dental implants^8–15^. The most important surface properties include topography, chemistry, surface charge, and wettability^16^. In addition to the implant material itself, the surface modification of the implant is an important basis for promoting the efficiency and stability of bone binding. At present, there are an increasing number of methods for surface modification of the implant. With the surface addition method, surface reduction method, surface bombardment method and surface oxidation method, many components, including Ca/P, Mg, Si, and Ta, with bone tissue formation induction properties can be added to the implant surface to promote the formation of bone tissue. In addition, some scholars added Ag, F, and Zn, which have antibacterial effects, to prevent infection^17–25^. In terms of design, many previous clinical studies have shown that a columnar or cone column design is the best choice. In China, frequently used oral implant systems include Branemark, STERI-OSS, NobelReplace, Straumann, Frialit-2, IMZ, Ankylos, Camlog, Xive, Lifecore, and Bicon. There are both advantages and disadvantages to each of the different systems.

However, although experts and scholars have performed many in vitro cytological studies investigating the surface modification of implants, to date, they have not designed, developed and verified an oral implant with good osseointegration in an in vivo model, and the domestic implants independently developed in China have not been recognized by most dental implant doctors and patients^26,27^. Therefore, in the current study, a new type of implant was developed and verified in vivo with beagle dogs, and the differences in the osseointegration properties between the novel implant and a commercial implant (Straumann SLActive) were observed and compared by using micro-CT and histology methods to identify the scientific basis of the osseointegration of the new implant.

## Materials and methods

### Implant design and surface characterization

Thirty-two novel implants (NIs) were manufactured from commercially available Ti-4 alloy (Ti-4; Trausim Medical Co., Ltd.). The implants were characterized by an identical cylindrical shape with a core diameter of 2.8 mm, an outer diameter of 4.2 mm, 4 miniature rings of 1.6 mm at the top, and double threads of 5.4 mm at the middle-lower part. The end of the implant was a spherical structure designed to avoid injury of the maxillary sinus mucosa. An internal four-angle fixing device prevented the abutment from easily rotating and inserting. All implants were first cleaned by ultrasonication, and then the samples were treated with 0.5% (w/v) hydrofluoric (HF) acid for 30 min. Immediately after acid treatment, the samples were rinsed with distilled water, dried and anodized for 30 min in an electrolyte-containing 0.5% (w/v) HF acid using a DC power (20 V) supply with a platinum electrode as the cathode^28^. Immediately after anodization, the samples were rinsed with deionized water and then sterilized in an autoclave. Next, the NIs were soaked in isotonic Hanks buffer (Xin Yu Co., Ltd., Shanghai, China). Sixteen commercial implants served as controls. Field-emission scanning electron microscopy (SEM; JSM- 6700F, JEOL) was used to observe the surface topography of the implants.

The structural designs of the NIs are shown in the pattern and physical map, including the miniature rings, double threads, spherical bottom, and four-angle fixing device (Fig. S1a). After the samples were etched and anodized, the size of micropores formed on the surface of the Ti-4 sample was the same as that of the controls; moreover, the distribution was uniform (Fig. S1b).

### Animals

This study was carried out under the approval of the Capital Medical University Animal Experiments and Experimental Animals Management Committee (Beijing, China. Approval No. AEEI-2017-104) and complied with the ARRIVE (Animal Research: Reporting of In Vivo Experiments) guidelines for preclinical animal studies (Appendix). All methods were carried out in accordance with relevant guidelines and regulations. Eight beagle dogs (1–2 years old, 15 kg) were obtained from the Institute of Animal Science, Chinese Agriculture University, China, and housed in individual cages in a controlled environment (20–25 °C, RH 40–60%). The animals were fed a soft dog-food diet (Science Diet, Hill’s Pet Co., Topeka, KS, USA) and had free access to water.

### Surgical procedures for tooth extraction and implant insertion

Animal surgery, including welfare-related assessments, interventions, and anesthesia, was performed as previously described^29^. In addition, veterinary assistance was used throughout all procedures, and all efforts were exerted to minimize pain. Briefly, all animals were preanesthetized with atropine sulfate (0.05 mg/kg intramuscular (IM) injection; Guangdong South China Pharmaceutical Co., Ltd., Guang Dong, China) and tiletamine/zolazepam (5 mg/kg IV; Zoletil 50; Virbac, Carros, France). Local anesthesia was achieved by infiltrating 1 mL of articaine (Produits Dentaires Pierre Rolland, France) containing epinephrine (1:100,000) into the mucosa at the surgical sites. The anesthesia was sustained with sevoflurane (Sevorane, Capital Medical University, Beijing, China). Care was taken to preserve the buccal, lingual, and lateral walls of the alveolar sockets. The teeth were carefully extracted without damaging the extraction sockets (Fig. S1c,d,e). Multimodal analgesia was used during the perioperative period. After the tooth extraction, Metacam 0.1 mg/kg [PO; Boehringer Ingemheim Co., Ridgefield, CT, USA], ketorolac 1 mg/kg [Toradol 30 mg, Shanghai Roche Pharmaceuticals Co., Ltd., Shang Hai, China], tramadol 1.7 mg/kg [Adolonta injectable, Grünenthal, Huayou medical group, Beijing, China], and buprenorphine 0.01 mg/kg [Buprex, Reckitt Benckiser Pharmaceuticals Limited, Berkshire, UK] were used to reduce pain. To prevent postoperative infection, amoxicillin (20 mg/kg PO; The United Laboratories Co., Ltd., Hong Kong, China) was administered for 6 days. Each dog was fed a liquid diet for 2 weeks, followed by a soft diet in a single cage. The extraction sites were allowed to heal for 3 months. In the next stage, the experimental and control implants were randomly implanted into the edentulate left and right jaw quadrants following the standard procedure with the same anesthesia and postoperative week care methods as previously described^10^. Postoperative pain control was performed as described above. To prevent postoperative infection, amoxicillin (20 mg/kg PO; The United Laboratories Co., Ltd., Hong Kong, China) was administered for 10 days. In addition, the dogs were treated with a plaque control regimen that included implant cleaning three times per week using a toothbrush and dentifrice. Each dog was fed a liquid diet for 2 weeks, followed by a soft diet in a single cage. There were 2 experimental implants and 1 control implant in every quadrant (Fig. S1f,g,h). At weeks 2, 4, 8, and 12, 2 dogs were euthanized by administering concentrated sodium pentobarbital IV (Euthasol, Delmarva Laboratories, Inc., Midlothian, VA, USA). Block sections were immediately collected from the implants, alveolar bone, and surrounding mucosa.

### Micro-CT evaluation

The specimens were scanned on a micro-CT system (Inveon, Siemens, Germany; 80 kV, 500 μA, 1500 ms exposure time) before mechanical and histological assessment. The regions of interest, including the trabecular compartment around the implant, were selected and defined as a cylinder with a radius of 0.5 mm from the implant surface and a length of 4 mm from the top of the implant. The trabecular thickness (Tb.Th), trabecular number (Tb.N), trabecular separation (Tb.Sp), bone surface/bone volume ratio (BS/BV), and bone volume/total volume ratio (BV/TV) were determined.

### Histological observation

The specimens were fixed with 4% formalin, decalcified with 10% ethylenediaminetetraacetic acid (pH 7.0) and dehydrated through a graded series of ethanol solutions and 100% acetone. Then, the specimens were embedded in methyl methacrylate, and tissue slides (25 μm) were prepared in the buccolingual direction parallel to the axis of the implants by an EXAKT 400CS grinding machine (Leica, Wetzlar, Germany). The slides were then stained with basic fuchsin, toluidine blue, and Goldner’s trichrome. Images were captured and analyzed by a light microscope (Olympus BH2 with S Plan FL2 lens, Tokyo, Japan) and a computer-digitized image analysis system (Leica Imaging System, Cambridge, England). Bone-to-implant contact (BIC) was calculated as the linear percentage of the interface with direct contact between bone and the implant to the total interface of the implant in the cancellous bone. For bone volume density (BD), the area fraction of the total peri-implant bone density as well as the newly formed peri-implant bone density was determined by calculating the percentage bone area inside the screw threads.

### Statistical analysis

All statistical calculations were performed with SPSS 16 statistical software (SPSS, Inc., Cary, NC, USA). Statistical significance was determined by independent-samples t test or analysis of variance. *p* values less than 0.05 were considered statistically significant: *p* < 0.05 (*), *p* < 0.001 (**), *p* < 0.0001 (****).

## Results

### Micro-CT evaluation showed similar peri-implant bone microstructures and bone density in both groups

**Figure 1:**
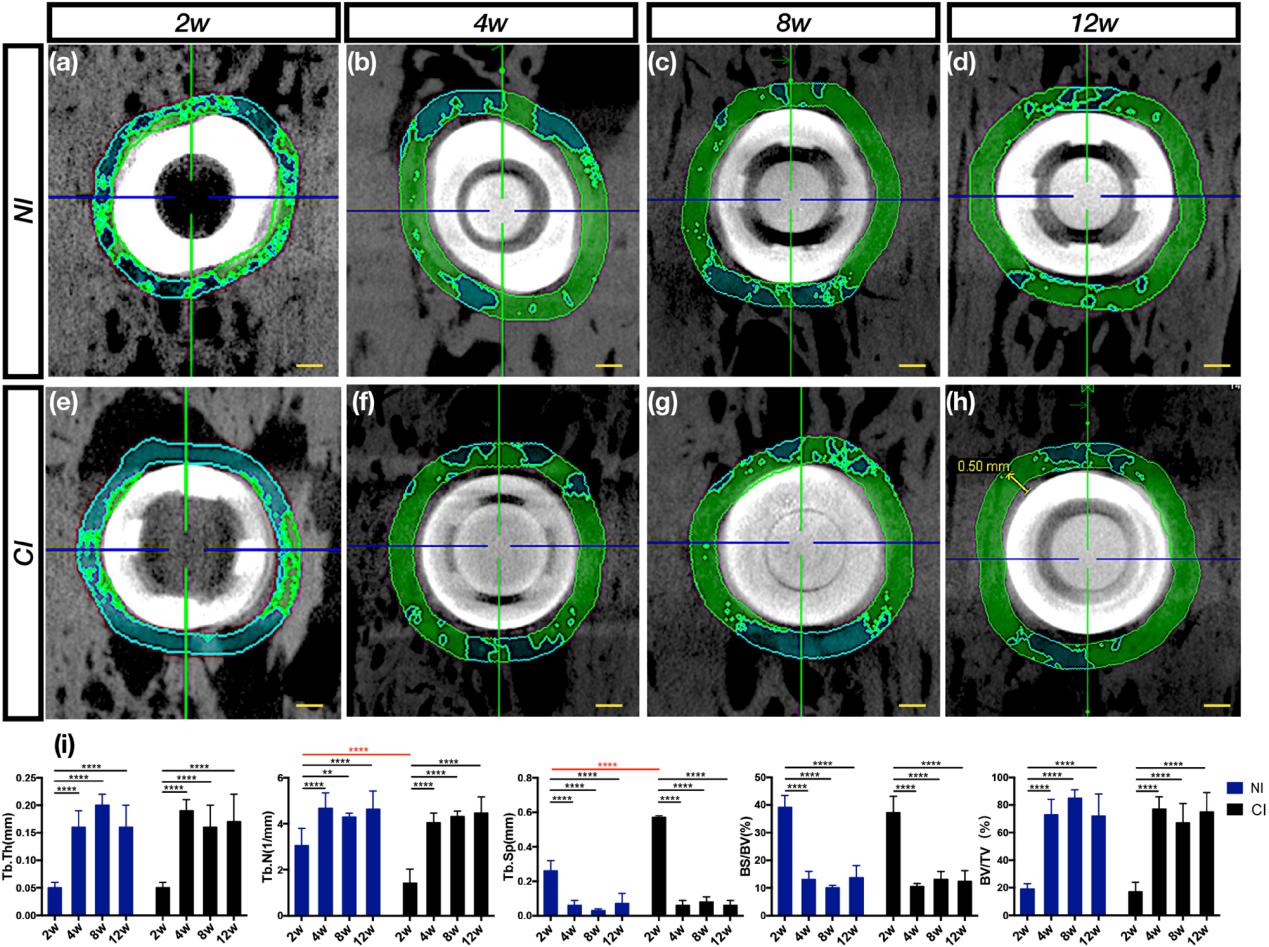
Osseointegration within a 0.5 mm cross-section from the surface and along the long axis top to 2 mm was measured through micro-CT. (**a**,**e**) The number of bone trabeculae around the two implants was low, indicating that the bone remodeling process was active and that there were fewer bone trabeculae in the CI than in the NI, suggesting that the surface of the treated CI was more active. (**b**–**d**,**f**–**h**) From the 4th to 12th weeks, the number of bone trabeculae in the two implants increased and stabilized. (**i**) Micro-CT evaluation of trabecular regions selected around the implant after 12 weeks of osseointegration. From the time of vertical perspective, osseointegration of the two implants was promoted from the 2nd to 4th weeks in terms of the Tb.Th, Tb.N, Tb.Sp, BS/BV and BV/TV. From the time of transverse perspective, bone modeling in the CI was more active than that in the NI in terms of the Tb.N and Tb.Sp at the 2nd week, but there were no differences at other times. Scale bars represent 0.5 mm.

The longitudinal profile of the implant was observed at 2 mm in the top axis (Fig. 1a–h). The time point at which bone remodeling was most active for the two implants was the second week after implantation. After 4 weeks, peri-implant bone remodeling tended to be stable. In addition, in the NI and control groups, there were significant changes over time between the 2nd and 4th weeks and no differences at other times. In contrast, the Tb.N increased, and the Tb.Sp decreased significantly over time in the experimental group (*p* < 0.0001). There were no statistically significant differences in the Tb.Th, BS/BV and BV/TV values between the two groups (Fig. 1i).

### NIs showed sustainable osseointegration in vivo by histological observation

**Figure 2:**
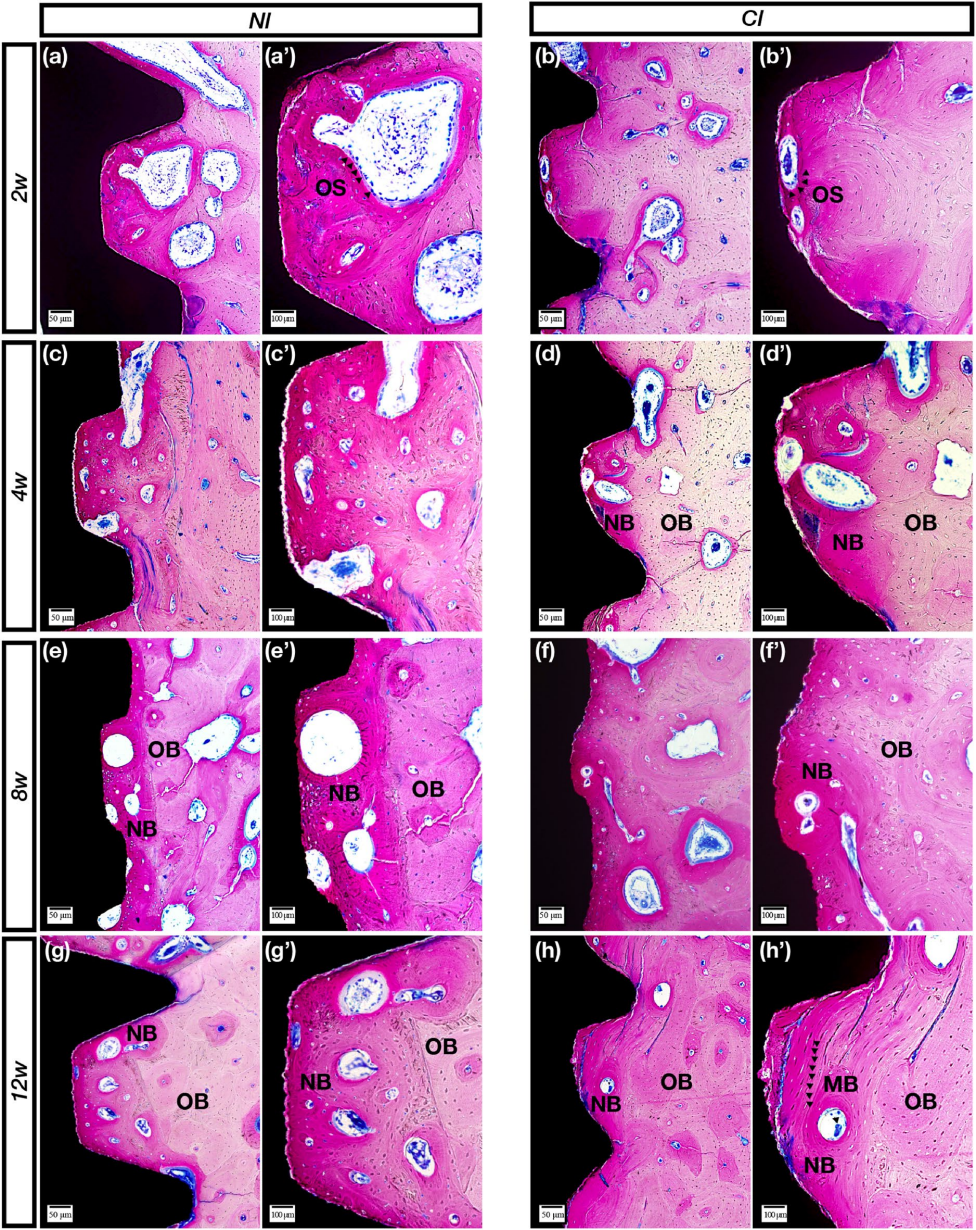
Methylene blue staining. (**a**,**a′**) Bone resorption lacuna of the NIs formed by some osteoclasts (black arrows) inside the thread in the 2nd week. (**b**,**b′**) Bone resorption lacuna of the CIs formed on the surface by some osteoclasts (black arrows) inside the thread in the 2nd week. (**c**,**c′**) On the 4th week, some bone resorption lacunae were formed on the surface of the NIs. (**d**,**d′**) In the 4th week, the bone resorption area of the control group increased, and deep pink new bone (NB) was observed. (**e**,**e′**) In the 8th week, almost no osteoclasts were observed, and extensive NB was observed between the threads. (**f**,**f′**) Almost no osteoclasts were observed in the experimental group, and the boundary between new and old bone became blurred. (**g**,**g′**) The boundary between new and old bone became more blurred for the NIs. (**h**,**h′**) Mineralized bone was observed on the surface of the CIs, and the NB became more mature in the 12th week. OS (osteoclasts); NB (new bone); OB (old bone); (MB) mineralized bone. Scale bars represent 50 μm (**a**–**h**) and 100 μm (**a′**–**h′**); original magnification: (**a**–**h**) × 10, (**a′**–**h′**) × 20.

**Figure 3:**
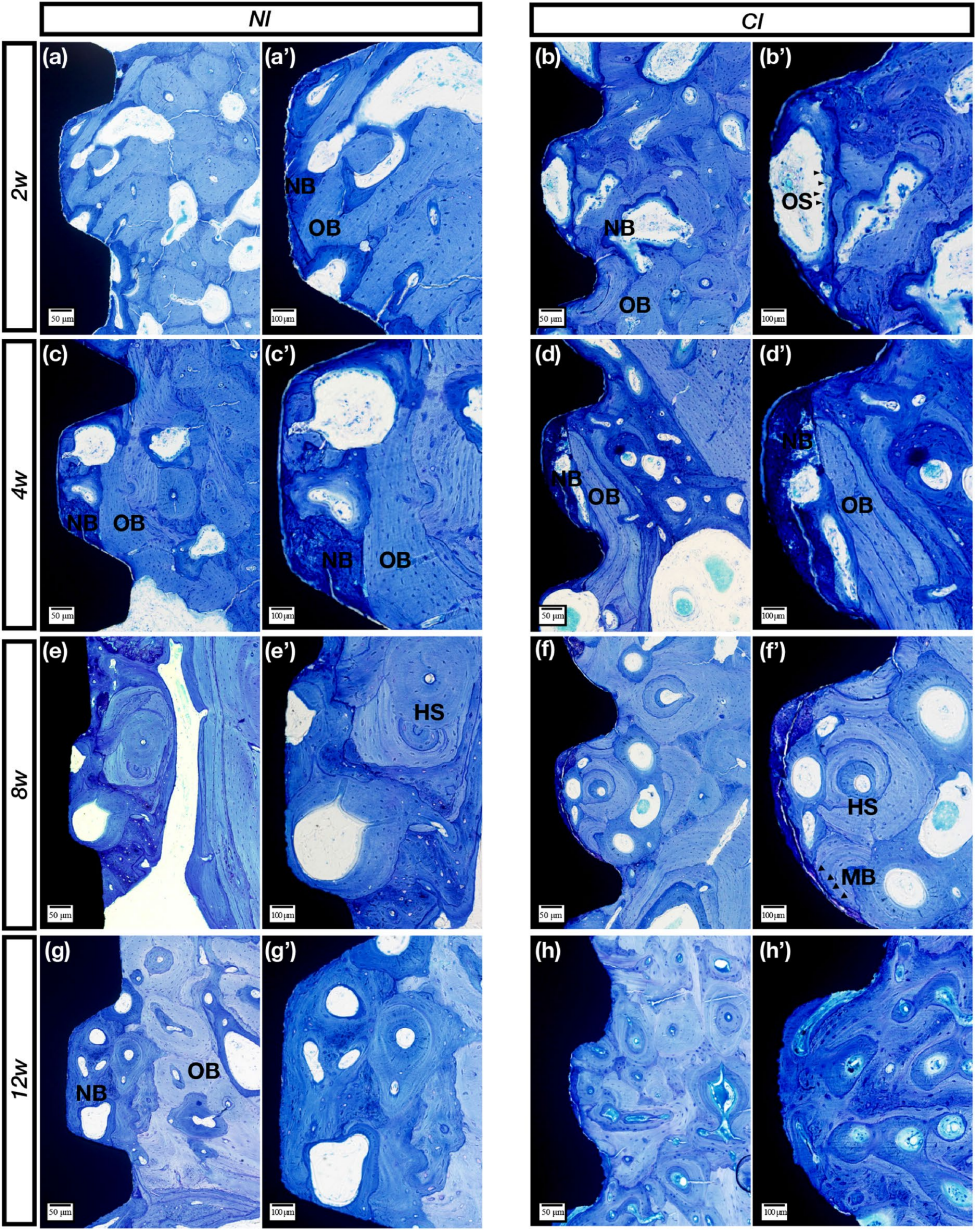
Toluidine blue staining. (**a**,**a′**) Clearly, the new and old bone of the NIs is alternately meshed in the threads in the 2nd week. (**b**,**b′**) For the control group, a large piece of irregular bone resorption sag appeared on the control implant surface due to the activities of some osteoclasts (black arrows). (**c**,**c′**) In the 4th week, some NB with partial bone resorption formed on the surface. (**d**,**d′**) In the 4th week, NB (dark blue staining) was evident on the CI surface. (**e**,**e′**) In the 8th week, the Haversian system (HS) was observed, and the cellular components disappeared. (**f**,**f′**) Mineralized bone (black arrows) deposited around the control implants, and NB gradually matured. (**g**,**g′**) The boundary between new and old bone became more blurred, and NB gradually matured after 12 weeks. (**h**,**h′**) The bone around the control implants was dominated by mature bone at 12 weeks. OS (osteoclasts); NB (new bone); OB (old bone); (HS) Haversian system; (MB) mineralized bone. Scale bars represent 50 μm (**a**–**h**) and 100 μm (**a′**–**h′**); original magnification: (**a**–**h**) × 10, (**a′**–**h′**) × 20.

**Figure 4:**
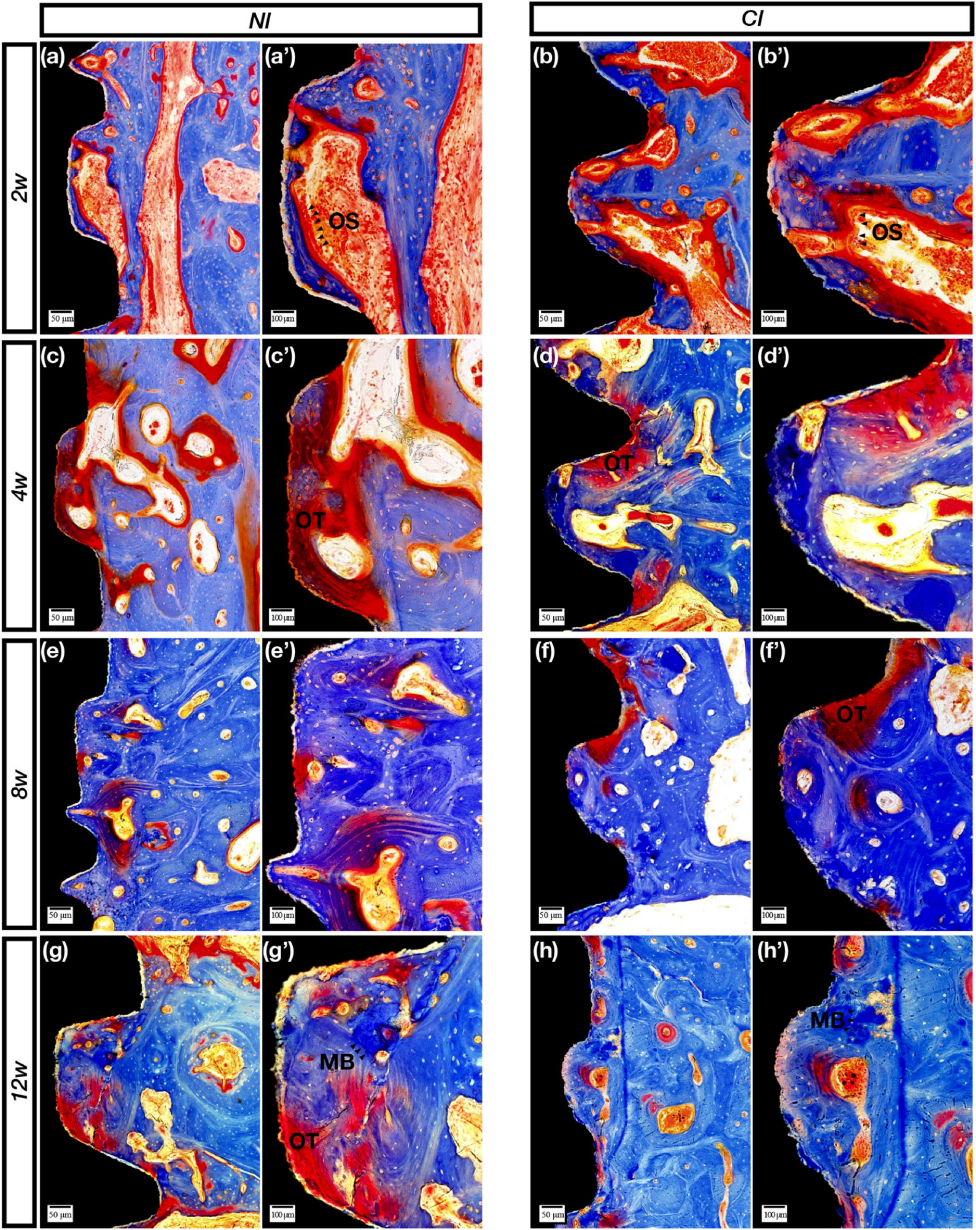
Goldner’s trichrome staining. (**a**,**a′**) Osteoclast (orange-yellow staining)-triggered bone remodeling activities at 2 weeks. (**b**,**b′**) Osteoclast-induced bone resorption appeared mostly on the control implant surface after 2 weeks. (**c**,**c′**) The osteoid (purple-red staining) was full of NI threads. (**d**,**d′**) Osteoid and partial NB were observed at 4 weeks. (**e**,**e′**) In the 8th week, interlaced bone, including the osteoid and NB, was observed. (**f**,**f′**) The boundary between new and old bone became more blurred (**g**,**g′**). Partially mineralized bone (bright blue staining), osteoid and mature bone were shown in the threads of the NIs after 12 weeks. (**h**,**h′**) Mature bone including mineralized regions. *OS* osteoclasts, *OT* osteoid, *MB* mineralized bone. Scale bars represent 50 μm (**a–h**) and 100 μm (**a′**–**h′**); original magnification: (**a**–**h**) × 10, (**a′**–**h′**) × 20.

The sections with basic fuchsin staining showed obvious bone remodeling activities from 2 to 4 weeks, and resorption was the main reaction (Fig. 2a–d′). The sections with toluidine blue staining showed that the boundaries between new and old bone became visibly clear at 2 weeks and gradually blurred after 4 weeks (Fig. 3a–d′). Furthermore, the sections stained with Goldner’s trichrome showed an enormous number of osteoclasts and a large amount of bone matrix around the implants at 2 weeks (Fig. 4a,b′). After 4 weeks, the amount of cell material gradually decreased, and the amount of bone matrix gradually increased (Fig. 4c,d′).

After 8 weeks, the sections with basic fuchsin staining showed that the osteoclasts gradually decreased in number, and the bone-resorbing lacunae gradually disappeared (Fig. 2e,f′). The sections with toluidine blue staining showed lavender-colored mineralized bone at 8 weeks (Fig. 3e,f′). The sections stained with Goldner’s trichrome showed that the number of osteoclasts was sparse, and purplish osteoid was present in the two groups (Fig. 4e,f′).

After 12 weeks, the sections with basic fuchsin staining showed that the bone around the two types of implants became denser, and the control implant had more mature bone (Fig. 2g,h′). Moreover, Haversian systems were found at 12 weeks in the control group (Fig. 3g,h′). Both osteoid and mineralized bone were observed, and there was no obvious boundary between new and old bone (Fig. 4g,h′).

**Figure 5:**
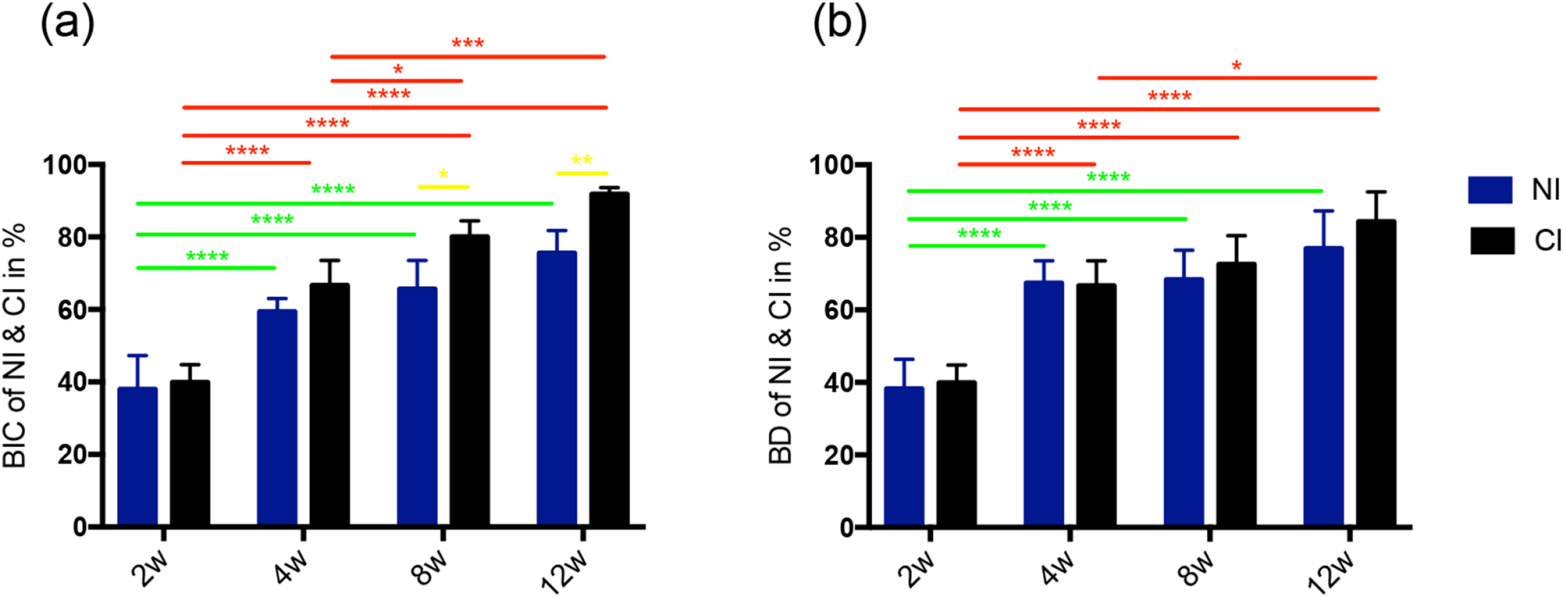
Histomorphometry results of BIC and BD. (**a**) From the longitudinal analysis of the time series, the BIC of the NIs was not significantly different from that at the fourth week. The BIC of the CIs showed a significant difference between the 2nd week and the other time points. A significant difference still existed between the 4th, 8th and 12th weeks. From the horizontal analysis of the time series, the BIC between the two implants was not significantly different from the 2nd to 4th weeks, but the BIC of CIs was higher at the 8th and 12th weeks. (**b**) From the longitudinal analysis of the time series, the BD of the NIs was not significantly different from that at the fourth week. The BD of the CIs showed obvious significant differences between the 2nd week and the other time points. A significant difference still existed between the 4th and 8th weeks. From the horizontal analysis of the time series, the BD between the two implants was not significantly different from the 2nd to 12th weeks. *p* values smaller than 0.05 were considered significant: *p* < 0.05 (*); *p* < 0.001 (**); *p* < 0.0001 (****). *BIC* bone-to-implant contact, *BD* bone density.

The longitudinal analysis of the time series showed that the changes in the direct BIC and BD of the NIs from the 2nd to 4th weeks were significantly different, but there were no significant differences after the fourth week. However, the horizontal analysis of the time series demonstrated that the BIC of the NIs was not significantly different from 2 to 12 weeks (Fig. 5a,b).

## Discussion

In the current study, NIs were modified by hydrofluoric acid and anodization. After HF acid etching and anodization, the increase in surface roughness at the micro/nanolevel might contribute to the improved implant osseointegration in osteoporosis patients^28,30–32^. Li et al. performed HF treatment on the titanium surface and showed that fluoride modification can produce many micro/nanostructured porous surfaces that are beneficial for osteoblast differentiation and gene expression^33^. Although Ti-4 has been able to achieve osteoblast adhesion and anticorrosion in some studies, the surface of the implant is still modified^34^. The NIs have excellent hydrophilic surfaces and in some studies, could accelerate the osseointegration process because the implant surface was continuously immersed in an isotonic solution that protects the implant surface from the carbonic acid and organic components naturally present in the atmosphere. As a result, the hydrophilic surface-bound hydroxylation/hydration groups showed an increased oxygen content and surface wettability, enhancing surface reactivity with the surrounding ions, amino acids and proteins in the tissue fluids.

Micro-CT is an important imaging method for evaluating the osseointegration effect of implants. Computed tomography can display the bone density around the implant and the microstructure of three-dimensional bone trabeculae. Compared with the traditional histological analysis method, it is less invasive and can directly calculate trabecular structural parameters through micro-CT analysis software, mainly including TB.N, Tb.Sp, Tb.Th, BS/BV and BV/TV^35–38^. The number of trabeculae, bone tissue maturity and bone mineral density were positively correlated with BS/BV and BV/TV. Tb.Sp is a negative indicator^39–41^. The results showed that TB.N and BV/TV increased over time following the implant implantation. In this study, the bone mineral density surrounding the implants in the two groups showed an upward trend, and the above indicators began to show significant differences between the two groups after 4 weeks. It can be speculated that the low osseointegration stability of the two groups was within 4 weeks, and the fourth week was probably the loading time of the implants in the two groups. The micro-CT analysis showed that at 2 weeks, the Tb.N in the NI group was higher than that in the control group and that the Tb.Sp was lower than that in the control group, indicating that the bone remodeling activity of the CIs was more active than that of the NIs at 2 weeks. This result was especially noticeable in the staining assay because the numbers of osteoclasts and bone resorption lacunae increased. From this point of view, the CIs were superior to the novel implants. Although the micro-CT data showed that both implants reached a relatively stable osseointegration state after 4 weeks, the Tb.Sp significantly decreased over time in the NI group. Further exploration and research are needed to improve the micro-bone quality and achieve good bone osteointegration.

Methylene blue staining is a common method for observing implant osseointegration and is especially useful for calculating the osseointegration rate and osseointegration density. In this study, there was no statistically significant difference in the osseointegration rate and density between the NIs and the control group. At 2 weeks, the osteoclasts were active in both implant groups. After 4 weeks, the bone remodeling process tended to be stable, but there did not seem to be a granular mineralized layer on the NI, which served as the control implant; moreover, this mineralized layer did not appear in every thread of the control implant. This was an interesting phenomenon that needs to be investigated in future work. Toluidine blue staining was used to clearly observe the boundaries between new and old bone. Before 4 weeks, the boundary was clear, especially at 2 weeks when the new bone was intertwined with the old bone. However, after 4 weeks, the bone was distributed in pieces, indicating that the density of the new bone reached a relatively stable value. Nonetheless, at 8 and 12 weeks, the degree of bone maturity and density in the thread in the experimental group was still lower than that in the control group, and although the density values did not significantly differ, the CIs still had a better osseointegration efficiency than the NIs. The results of Goldner’s trichrome staining clearly showed the whole process of bone healing, including bone tissue resorption by the osteoclasts, bone matrix secretion, bone matrix mineralization to form osteoid, and replacement of new and old bone. In the current study, at 2 weeks, a large number of orange-colored osteoclasts were observed in both groups. After 4 weeks, there was a large amount of osteoid, and more appeared on the new implants, indicating that the osseointegration efficiency was lower than that in the control group. After 12 weeks, the two groups showed clear and bright blue mineralization structures; however, the bone structure in the control group seemed more mature. The above analysis showed that although the osseointegration efficiency of the NIs was not the same as that of the CIs, the NIs could still achieve essential osteointegration.

## Conclusions

In summary, through histological and micro-CT studies of the first dental implant independently developed in China, it was found that the Tb.Th, Tb.N, Tb.Sp, BS/BV, BV/TV and BD of the NIs basically matched those of commercial CIs from 2 to 8 weeks after implantation and that the NIs basically achieved a stable state of osseointegration within 4 weeks. However, the BIC of the NIs was still lower than that of the CIs at 8 and 12 weeks after implantation, indicating that the surface treatment technology of the NIs still needs further study. In addition, the long-term stability of osseointegration or the risk of marginal bone resorption still needs to be further studied.

## Supplementary Information

Supplementary Figure S1.

Supplementary Legend.
