# Supplementary figures and images for: Osseointegration of a novel dental implant in canine
Source: Sci Rep. 2021 Feb 22;11:4317. doi: 10.1038/s41598-021-83700-4 (PMC7900171; doi:10.1038/s41598-021-83700-4)

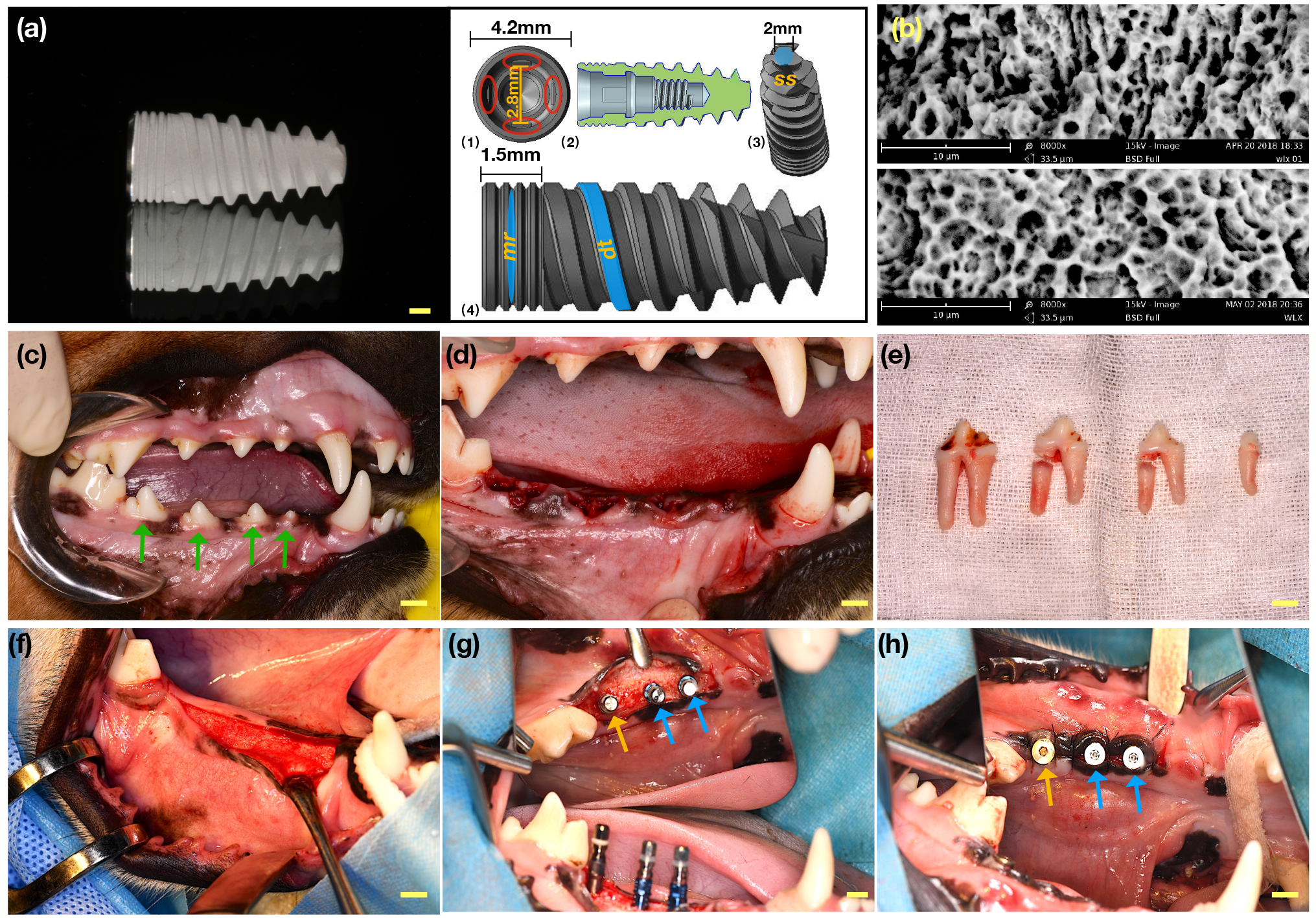

Supplement: Supplementary file 1 — Supplementary Figure S1. [file 41598_2021_83700_MOESM1_ESM.tif]
