# Supplementary material for: Osseointegration of a novel dental implant in canine
Source: Sci Rep. 2021 Feb 22;11:4317. doi: 10.1038/s41598-021-83700-4 (PMC7900171; doi:10.1038/s41598-021-83700-4)
Supplement: Supplementary file 2 — Supplementary Legend. [file 41598_2021_83700_MOESM2_ESM.docx]

Title: **Osseointegration of a novel dental implant in canine**

Running title: Osseointegration of a novel dental implant

Keywords: osseointegration, Chinese implant, dental implant, histomorphometric study, beagles

Lingxiao Wang^1#^, Zhenhua Gao^1#^, Yucheng Su^2,3^, Qian Liu^3^, Yi Ge^2*^, Zhaochen Shan^1*^

^1^Outpatient Department of Oral and Maxillofacial Surgery, School of Stomatology, Capital Medical University, Beijing 100050, P. R. China

^2^Department of Stomatology, Chinese Academy of Medical Science & Peking Union Medical College, Beijing, 100032, P. R. China

^3^Beijing Citident Stomatology Hospital, Beijing, 100032, P. R. China

^#^These authors contributed equally to this article

*Co-corresponding author:

Yi Ge, M.D.

Department of Stomatology, Peking Union Medical College Hospital, No. 41 Damucang Hutong, Xicheng District, Beijing, 100032, P. R. China;

Tel: 13801200189

E-mail: geyi69@163.com

Zhaochen Shan, M.D.

Outpatient Department of Oral and Maxillofacial Surgery, School of Stomatology, Capital Medical University, Tian Tan Xi Li No. 4, Beijing 100050, P. R. China;

Tel: 13718488361

E-mail: shanzhch629@163.com

E-mail of coauthors:

Lingxiao Wang: lingxiaoccmu@163.com

Zhenhua Gao: gaozhenhua21@163.com

Yucheng Su: yuchengsu@163.com

Qian Liu: qianliu1210@163.com

Yi Ge: geyi69@163.com

Zhaochen Shan: shanzhch629@163.com

Supplementary Materials

Figure S1. Structural design, surface topography and surgical operation of the NIs. (a) Implant prototype. Cylindrical shape with 4 miniature rings of 1.5 mm and double thread of 6 or 8 mm at the middle-lower part; a core diameter of 2.8 mm, an outer diameter of 4.2 mm and a four-angle fixing device; the end of the implant is a 2 mm spherical structure. (b) The SEM view (amplified 8000 times) of the NI and SR implant surface. (c) Four mandibular premolars (green arrows). (d) Microdamage alveolar fossae. (e) Complete extraction of teeth. (f) Flap. (g) Implantation of NIs (blue arrows) and controls (yellow arrows). (h) Suture. Scale bars represent 1 mm (a), 10μm (b), and 1 cm (c-h).
